# Invasive candidiasis presenting multiple pulmonary cavitary lesions on chest computed tomography

**DOI:** 10.1186/s40248-015-0009-0

**Published:** 2015-03-20

**Authors:** Yuichiro Yasuda, Kazunori Tobino, Mina Asaji, Yoshikazu Yamaji, Kosuke Tsuruno

**Affiliations:** Department of Respiratory Medicine, Iizuka Hospital, 3-83 Yoshiomachi, Iizuka, Fukuoka, 820-0018 Japan; Department of Respiratory Medicine, Juntendo University, School of Medicine, 2-1–1 Hongo, Tokyo, Bunkyo-Ku 113-8421 Japan

**Keywords:** Canididemia, Computed tomography, Invasive candidiasis, Pulmonary cavity

## Abstract

We herein report a case of invasive candidiasis presenting rare findings on chest computed tomography (CT). The chest CT scan showed multiple small cavitary lesions and nodules with surrounding ground-glass opacity, and also bilateral pleural effusion. Although this CT finding is thought as specific for pulmonary aspergillosis, two sets of blood culture specimens were drawn which yielded *Candida albicans* in our case. Antifungal therapy was started and the chest CT findings showed a remarkable improvement. To our knowledge, this is the first case report describing multiple pulmonary cavitary lesions in invasive candidiasis.

## Background

It has been reported that invasive candidiasis is rare but associated with considerable mortality in immunocompromised or critically ill patients. The most common chest computed tomography (CT) findings of invasive candidiasis have been reported as multiple bilateral nodules often associated with air-space consolidation [1]. Here, we present a rare case of invasive candidiasis presenting multiple pulmonary cavitary lesions on chest CT. To our knowledge, this is the first case report of invasive candidiasis with this finding in English literatures.

## Case presentation

**Figure 1:**
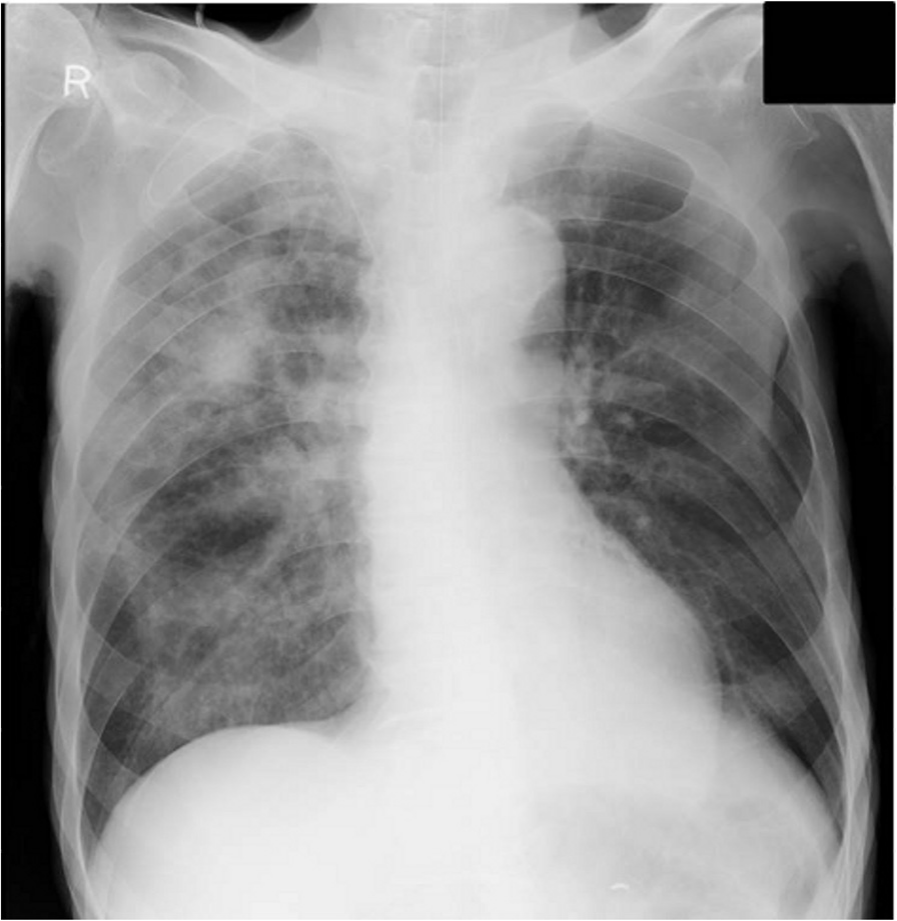
**Multiple nodules and ground-glass opacities.** The chest x-ray showed multiple nodules in the right upper lung field, and mixed ground-glass and airspace opacities in the entire right lung.

**Figure 2:**
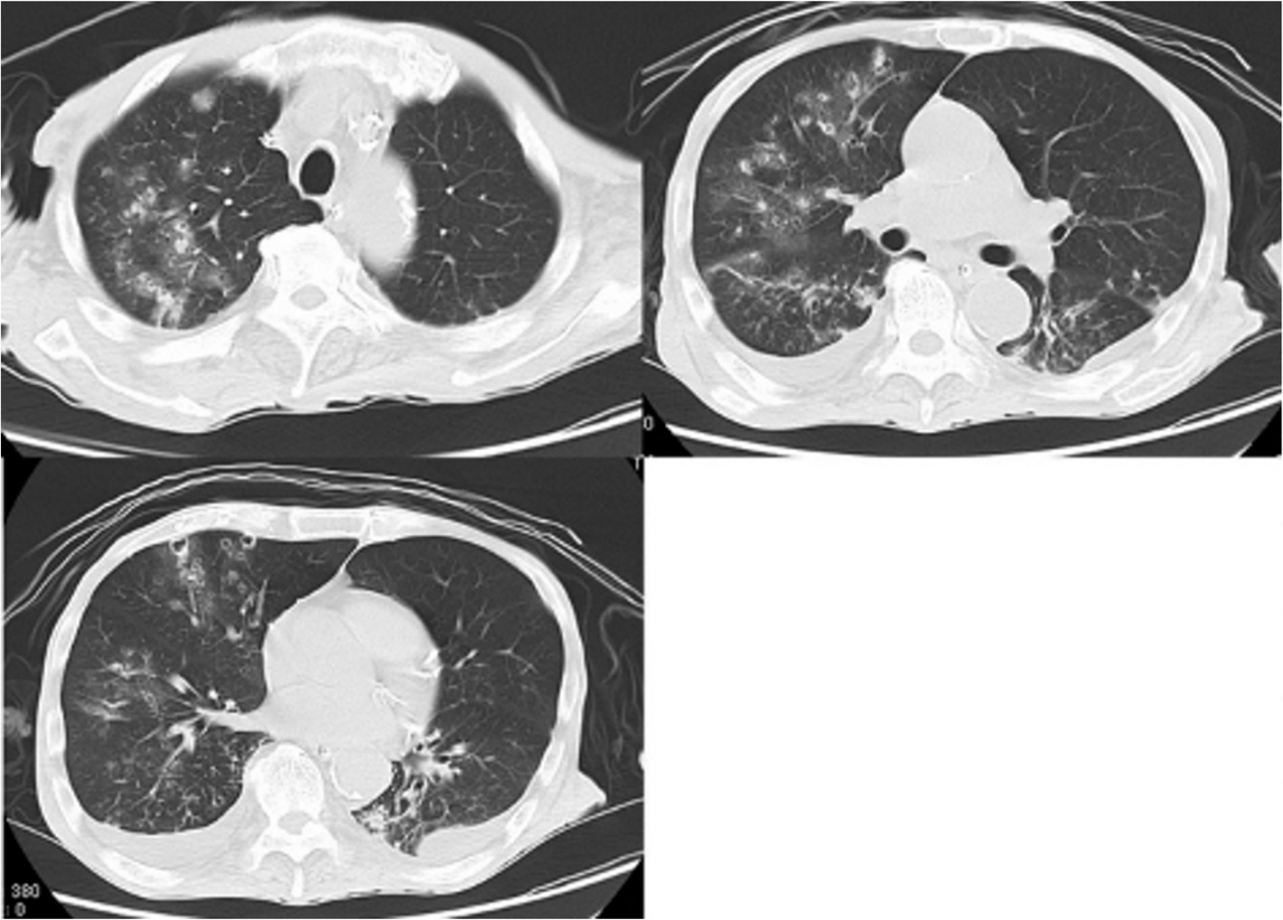
**Multiple small cavitary lesions and nodules.** Chest CT images obtained on the 14^th^ day of admission showed multiple small cavitary lesions and nodules surrounded by ground-glass opacity, and also bilateral pleural effusion. These lung abnormalities seemed to be in a peribronchovascular distribution.

**Figure 3:**
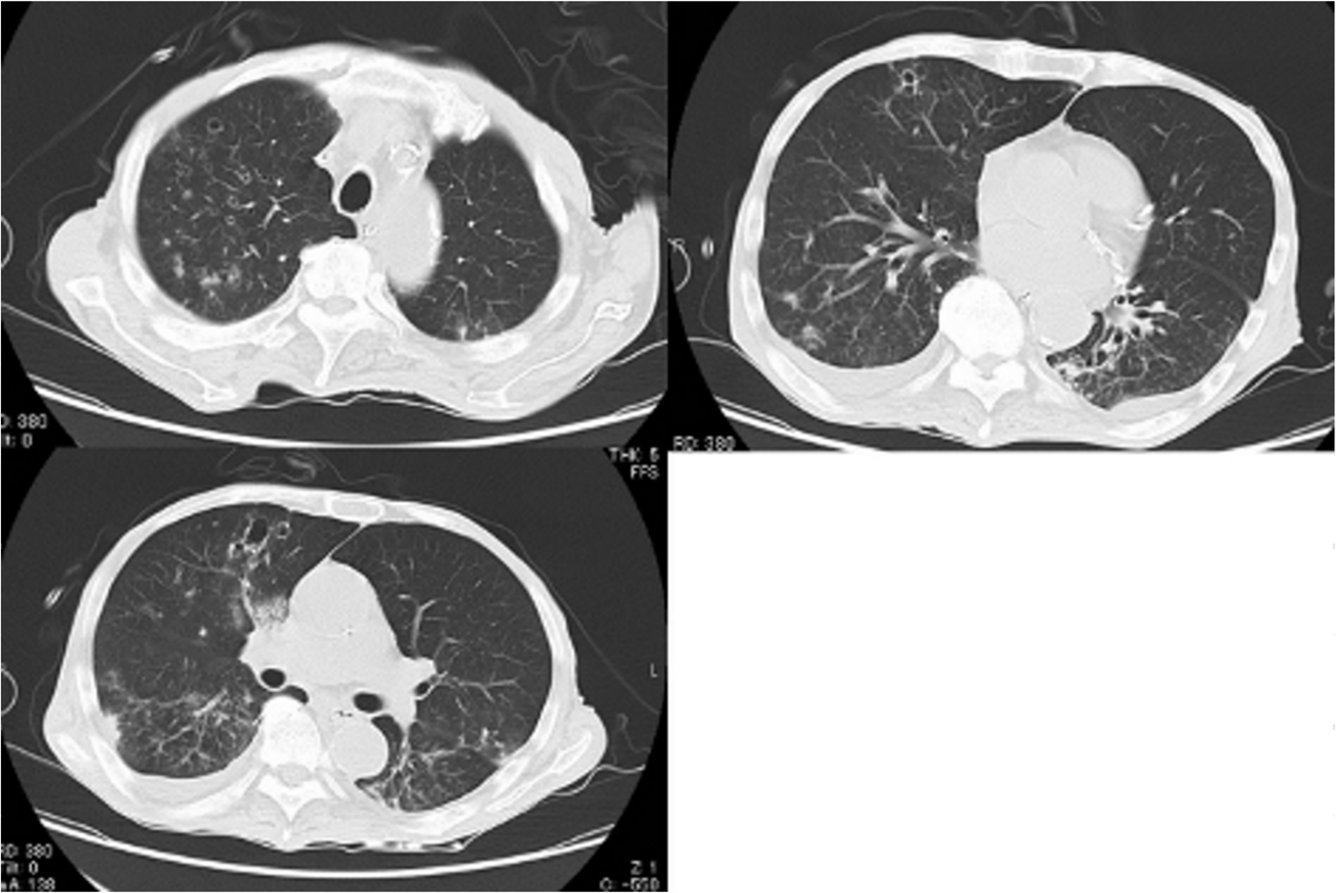
**Chest CT after antifungal therapy.** Chest CT images obtained on the 29^th^ day of admission showed that multiple small cavitary lesions, nodules and ground-glass opacity had improved after antifungal therapy.

An 80-year-old man was admitted to our hospital for the treatment of small bowel obstruction. Initial management involved nasogastric tube insertion and fluid resuscitation with central venous (CV) catheters. His small bowel obstruction did not resolve, and on the 8^th^ day, he presented fever and hypoxemia. He had a history of stage IV chronic kidney disease secondary to hypertensive nephrosclerosis for 15 years, and distal gastrectomy for gastric cancer 20 years before. He did not have smoking history and risk factors for HIV infection, and drank alcoholic beverages occasionally. Physical examination revealed poor oral hygiene only. The chest x-ray revealed multiple nodules in the right upper lung field, and also mixed ground-glass and airspace opacities in the entire right lung (Figure 1). The chest CT scan showed multiple small cavitary lesions and nodules surrounded by ground-glass opacities, and also bilateral pleural effusion (Figure 2). Examination of sputum showed no predominant pathogen and no acid-fast organisms on staining. Laboratory tests revealed elevated serum β-D-glucan (483 pg/ml, normal, < 20 pg/ml) positive serum Candida antigen latex agglutination test, and negative serum Aspergillus galactomannan antigen test. Two sets of blood culture specimens were drawn on the 8^th^ day which yielded *Candida albicans*. Transbronchial biopsy and bronchial washings of the cavitary lesion in the right upper lobe were performed, however, non-specific inflammation of the lung tissue without any bacteria was revealed. Moreover, transbronchial biopsy did not reveal aspergillus hyphae. The patient was diagnosed as affected with invasive candidiasis. Therefore, potentially contaminated CV catheter was removed and antifungal therapy with intravenous fluconazole was started. The patient became afebrile after the 3^rd^ day of the initiation of antifungal therapy, and blood culture of the same day did not yield any organisms. The treatment was continued for three weeks, and on the 15^th^ day of antifungal therapy the chest CT findings showed a remarkable improvement (Figure 3).

*Candida* spp. exist as normal flora of the human skin, oropharynx, lower gastrointestinal tract, and genitourinary system. *Candida* spp. are now one of the most common causes of nosocomial blood stream infections worldwide [2]. Invasive candidiasis is a fungal infection that can occur when *Candida* spp. enter the bloodstream. Once the fungus is in the bloodstream, it can spread to other parts of the body and cause infection. There are risk factors for invasive candidiasis, such as central venous catheter, surgical procedure, acute renal failure, disseminated intravascular coagulopathy, parenteral nutrition and the use of broad-spectrum antibiotics [3]. In our case, parenteral nutrition with CV catheter was thought to be the risk factor for invasive candidiasis. Moreover, small bowel obstruction was also thought to be the risk factor because the protective mechanisms of intestinal mucosa were thought to be broken.

The few available studies on this topic indicate that pulmonary manifestations of invasive candidiasis are seen in no more than 0.2 to 8.0% of at-risk ICU patients and cancer patients [2]. As with other opportunistic mycoses, sputum cultures are unreliable for diagnosis, because the organism frequently colonizes in the upper airways, and a definitive diagnosis requires culture of *Candida* from blood, normally sterile organ or body cavity [3,4]. We could diagnose our case as invasive candidiasis by the positive blood culture and chest CT findings. The most common chest CT findings were reported as multiple bilateral nodules often associated with air-space consolidation, however these findings are nonspecific and the differentiation from other fungal infections (especially aspergillosis) is difficult. In pulmonary fungal infections cavitation has been considered to represent concomitant bacterial infection or hemorrhagic lung infarcts, and the surrounding ground-glass or air-space opacity has been considered to represent a mixture of edema and hemorrhage [2]. The multiple cavitary lesions seen in our patient were thought to represent septic pulmonary infarcts due to blood-stream infection of *Candida albicans*, and this CT finding is rare in invasive candidiasis. It was reported that cavitary lesions were less common in invasive candidiasis (4%) than in aspergillosis (16%) [5], and, to our knowledge, this is the first case report describing multiple pulmonary cavitary lesions in invasive candidiasis.

## Conclusions

In conclusion, multiple pulmonary cavitary lesions are rare CT manifestations of invasive candidiasis. This CT finding is thought as specific for pulmonary aspergillosis, however, we should also consider this finding in invasive candidiasis occurring in patients who have risk factors. We believe that our case will be helpful to the understanding and recognition of the spectrum of this rare condition.

## Consent

Written informed consent was obtained from the patient for publication of this Case report and any accompanying images. A copy of the written consent is available for review by the Editor-in-Chief of this journal.

## Abbreviations

CT: Computed tomography
CV: Central venous
